# Interpretable Sensor Change Detection via Conditional Cauchy–Schwarz Divergence

**DOI:** 10.3390/s26061791

**Published:** 2026-03-12

**Authors:** Wenyu Wang, Yuan Shen, Yao Ni, Wangyu Wu

**Affiliations:** 1 Department of Statistical Science, University College London, London WC1E 6BT, UK; uceeww2@ucl.ac.uk; 2 Meta Platforms Inc., Menlo Park, CA 94025, USA; yuans511@meta.com; 3 School of Integrated Circuit Engineering, Guangdong University of Technology, Guangzhou 510006, China; 4 School of Computer Science, University of Liverpool, Liverpool L69 3DR, UK

**Keywords:** change point detection, sensor networks, Cauchy–Schwarz divergence, interpretability, industrial process monitoring

## Abstract

Detecting distributional changes in multivariate sensor networks is a fundamental task for monitoring complex systems such as industrial processes, structural health monitoring, and large-scale Internet of Things infrastructures. Despite significant progress, most existing change-point detection methods either operate on high-dimensional observations in a black-box manner or provide limited insight into how inter-sensor dependencies evolve over time, thereby restricting their practical utility in safety-critical applications. In this work, we propose an interpretable change detection framework based on the Cauchy–Schwarz (CS) divergence. By extending CS divergence to conditional distributions over sensor variables, the proposed method detects distributional shifts through changes in sensor-wise conditional relationships. This design enables reliable change detection while simultaneously providing transparent sensor-level explanations of detected changes. Extensive experiments on synthetic data, generic multivariate sensor time series, and a large-scale industrial process benchmark demonstrate that the proposed method achieves competitive or superior detection performance compared to representative baselines, while offering fine-grained interpretability for practical sensor monitoring systems.

## 1. Introduction

Quantifying changes in probability distributions is a fundamental problem in statistics and machine learning, with applications ranging from anomaly detection and system monitoring to representation learning and generative modeling [1,2]. In many real-world scenarios, multivariate time series are generated by non-stationary processes whose underlying distributions evolve over time due to environmental variations, system degradation, or external interventions. For example, in industrial automation, subtle changes in conditional dependencies among sensors may indicate early-stage equipment degradation; in healthcare monitoring, distributional shifts in physiological signals may signal abnormal patient states; and in cybersecurity, traffic distribution changes may reveal emerging attacks.

When such distributional shifts occur, predictive models trained on historical data may suffer significant performance degradation, potentially leading to unreliable decisions in safety-critical domains such as industrial automation, healthcare monitoring, and large-scale sensor networks. Therefore, accurately detecting and quantifying distributional changes is essential for ensuring robustness, reliability, and trustworthiness in modern machine learning systems.

In this context, change-point detection (CPD) provides a principled framework for identifying time instants at which the data-generating process undergoes significant shifts [3]. A large body of existing CPD methods focuses on comparing probability distributions across temporal windows using divergence measures such as the Kullback–Leibler divergence, maximum mean discrepancy, or Wasserstein distance [4,5]. While effective at signaling the presence of a change, these approaches typically operate at the level of joint or marginal distributions and offer limited interpretability. In practice, however, detecting that a change has occurred is often insufficient: practitioners must also determine which variables are responsible for the observed change, particularly in high-dimensional systems.

Recent advances have begun to address this limitation by incorporating feature-wise or dependency-aware perspectives into change detection. Examples include density-ratio-based methods [6], kernel-based two-sample tests [7,8], and classifier-based approaches that leverage representation learning to enhance detection power [9]. More recently, interpretable formulations have been explored through conditional distribution tests that aim to localize which features have shifted over time [10]. Despite this progress, a unified framework that simultaneously offers distributional sensitivity, computational efficiency, and intrinsic interpretability in high-dimensional settings remains limited.

Large-scale sensor networks exhibit complex inter-variable dependencies arising from physical couplings and shared operational factors. In such systems, distributional changes often manifest as localized shifts in conditional relationships rather than global deviations of the joint distribution. This observation motivates an interpretable change detection framework based on conditional distribution analysis.

Accordingly, we characterize distributional changes through variable-wise conditional relationships and employ the classical Cauchy–Schwarz (CS) divergence [11,12] and its conditional extension [13] as information-theoretic measures of temporal discrepancies. Building on this idea, we develop an interpretable change detection framework that evaluates conditional distribution shifts at the level of individual variables. By decomposing discrepancies across sensors, the proposed approach enables localization of the variables whose dependency structures drive detected changes. Experiments on synthetic data, generic multivariate sensor time series, and a large-scale industrial process benchmark demonstrate competitive detection performance while providing sensor-level interpretability.

To summarize, this work makes the following contributions:

- First, we formulate interpretable change-point detection in multivariate sensor time series from a conditional distribution perspective, explicitly linking distributional changes to shifts in sensor-wise conditional relationships.
- Second, we introduce a conditional form of the classical Cauchy–Schwarz divergence as an information-theoretic tool to quantify conditional distributional discrepancies in a nonparametric and differentiable manner.
- Third, we develop an interpretable change detection framework that decomposes conditional discrepancies across individual sensors, enabling fine-grained localization of the sources of change without relying on black-box learning models.

## 2. Related Work

### 2.1. Time Series Change Point Detection

Change point detection (CPD) aims to identify time instants at which the statistical properties of a time series undergo significant changes. It is a classical problem in statistics and machine learning with applications ranging from process monitoring and anomaly detection to finance, biomedical signal analysis, and large-scale sensor networks. Depending on whether data are processed retrospectively or sequentially, CPD methods can be broadly categorized into offline and online settings. We refer interested readers to the comprehensive survey by Truong et al. [3].

From a distributional perspective, many CPD methods formulate the problem as detecting discrepancies between probability distributions across two temporal segments. Given a multivariate time series ${\left\{x_{t}\right\}}_{t=1}^{T}$ with $x_{t}\in R^{m}$, a change point at time $t_{0}$ is declared if the divergence between the distributions of observations from two adjacent windows exceeds a predefined threshold:

(1) $$D\left(p\left(x_{t_{0}-W:t_{0}-1}\right),\;p\left(x_{t_{0}:t_{0}+W-1}\right)\right)\geq \delta ,$$

where *W* denotes the window size and D(·, ·) is a suitable measure of distributional discrepancy. This formulation is flexible and model-agnostic, and has become a dominant paradigm for nonparametric change detection in high-dimensional time series.

Early distributional approaches estimate divergences directly via nonparametric density estimation, such as kernel density estimators (KDEs) [14]. However, KDE-based divergence estimation becomes unreliable in high-dimensional settings due to the curse of dimensionality. To address this issue, density-ratio estimation methods have been proposed, which estimate the ratio of two distributions without explicitly estimating the individual densities. Representative examples include the Kullback–Leibler importance estimation procedure (KLIEP) [15] and relative unconstrained least-squares importance fitting (RuLSIF) [6]. These methods are computationally efficient and have been widely adopted in practical change detection systems, particularly in online and streaming scenarios.

Another prominent line of work formulates CPD as a two-sample hypothesis testing problem using kernel methods. The maximum mean discrepancy (MMD) [16] compares distributions via their mean embeddings in reproducing kernel Hilbert spaces, thereby avoiding explicit density estimation. MMD-based detectors are flexible, nonparametric, and applicable to a wide range of data types, and have been successfully applied to change detection in time series [7,8]. More recently, kernel change-point detection has been integrated with deep models to enhance detection power in complex and highly structured data distributions [9,17].

Recent efforts have begun to address the interpretability limitations of conventional CPD methods by explicitly localizing which features or dependencies are responsible for detected distributional shifts. A representative line of work formulates interpretability in terms of conditional distribution changes. Kulinski et al. [10] formalized feature shift detection via conditional distribution tests, framing interpretability as identifying dimensions whose conditional distributions $p(x^{i}|x^{-i})$ change over time. Nie and Nicolae [18] study detection and localization of conditional distribution shifts from a hypothesis testing perspective. Related perspectives on conditional dependence and invariance have also been explored in the context of causal discovery [19,20].

In parallel, several approaches rely on classifier-based two-sample tests to detect distributional changes [21,22]. While often powerful in high-dimensional settings, such methods typically require post hoc attribution techniques to infer which features drive the detected shift, and their explanations may depend strongly on model inductive biases. Another complementary line of research characterizes changes through variations in dependency or graph structures, such as mutual information matrices or dynamic networks [23,24].

### 2.2. The Cauchy–Schwarz Divergence and Its Conditional Extension

Before introducing our change detection framework, we briefly review the CS divergence and its conditional extension, which provide fundamental information-theoretic tools for measuring distributional discrepancies and serve as building blocks of the proposed method.

A line of work initiated by Principe and colleagues in the early 2000s proposed divergence measures constructed from classical inequalities in functional analysis [11,12]. A representative example is the CS divergence, which is rooted in the Cauchy–Schwarz inequality applied to probability density functions. Given two densities p(x) and q(x) defined on a common domain, their $L^{2}$ inner product satisfies

(2) $${\left(\int p(x)\;q(x)\;dx\right)}^{2}\leq \left(\int p{\left(x\right)}^{2}\;dx\right)\left(\;\int q{\left(x\right)}^{2}\;dx\right),$$

where equality holds if and only if *p* and *q* are linearly dependent in $L^{2}$. This inequality motivates measuring distributional discrepancy by comparing the cross-information term ∫p(x)q(x)dx against the self-information terms $\int p{\left(x\right)}^{2}dx$ and $\int q{\left(x\right)}^{2}dx$. Accordingly, the CS divergence is defined as

(3) $$D_{\mathrm{CS}}\left(p;q\right)=-\log\frac{{\left(\int p(x)q(x)\;dx\right)}^{2}}{\left(\int p{\left(x\right)}^{2}\;dx\right)\left(\int q{\left(x\right)}^{2}\;dx\right)}.$$

An equivalent view is that $D_{\mathrm{CS}}\left(p;q\right)$ is the negative logarithm of a normalized squared correlation (cosine similarity) between *p* and *q* in the Hilbert space $L^{2}$, and thus it decomposes naturally into quadratic (self) and cross terms involving *p* and *q*.

The CS divergence enjoys several properties that make it attractive for statistical signal processing and change detection. For certain distribution families, such as mixtures of Gaussians, the integrals in Equation (3) admit closed-form expressions [25]. More generally, it can be estimated efficiently using nonparametric density estimators. In contrast to the Kullback–Leibler (KL) divergence, the formulation in Equation (3) avoids explicit density-ratio estimation and logarithms of densities, which often improves numerical stability in practice [26].

Given i.i.d. samples ${\left\{x_{p,i}\right\}}_{i=1}^{M}$ and ${\left\{x_{q,j}\right\}}_{j=1}^{N}$ drawn from p(x) and q(x), respectively, the integrals in the CS divergence can be approximated using kernel density estimation (KDE). Employing a Gaussian kernel $G_{\sigma }\left(x\right)={\exp(-∥x∥}^{2}/2\sigma^{2})$ with kernel width σ, the CS divergence admits an empirical estimator of the form:

(4) $$\begin{array}{cc} {\hat{D}}_{\mathrm{CS}}\left(p;q\right)= & \log\;\left(\frac{1}{M^{2}}\sum_{i,j}G_{\sigma }\left(x_{p,i}-x_{p,j}\right)\right)+\log\;\left(\frac{1}{N^{2}}\sum_{i,j}G_{\sigma }\left(x_{q,i}-x_{q,j}\right)\right)\\ & \;-2\log\;\left(\frac{1}{MN}\sum_{i,j}G_{\sigma }\left(x_{p,i}-x_{q,j}\right)\right). \end{array}$$

Importantly, the estimator is nonparametric, meaning that it does not impose any predefined parametric assumptions (e.g., Gaussianity) on the underlying data distributions. This property is particularly relevant in our setting. In multivariate sensor monitoring and industrial process analysis, the underlying data-generating mechanisms are often nonlinear, partially unknown, and difficult to model accurately using parametric distributions (e.g., Gaussian or linear models). A nonparametric formulation enables direct comparison of empirical window-based distributions without risking model misspecification, thereby improving robustness in complex real-world systems.

Note that the consistency of the underlying KDE-based estimator follows from standard KDE theory (see Appendix B.4 of [27] for a detailed proof), and therefore the CS divergence estimator inherits these guarantees.

The CS divergence can be naturally extended to conditional distributions. Let y∈R and $x\in R^{m-1}$ denote two sets of random variables, and consider two conditional distributions p(y∣x) and q(y∣x). The conditional CS divergence is defined as [13]:

(5) $$\begin{array}{cc} D_{\mathrm{CS}}\left(p\left(y|x\right);q\left(y|x\right)\right)= & -2\log\;\left(\int_{x}\int_{y}p\left(y|x\right)q\left(y|x\right)\;dx\;dy\right)\\ & +\log\;\left(\int_{x}\int_{y}p{\left(y|x\right)}^{2}\;dx\;dy\right)+\log\;\left(\int_{x}\int_{y}q{\left(y|x\right)}^{2}\;dx\;dy\right), \end{array}$$

The conditional CS divergence also admits an efficient empirical approximation based on KDEs, analogous to the unconditional case. This estimator enables practical evaluation of discrepancies between conditional distributions directly from samples, which is essential for interpretable analysis of multivariate distributional changes.

Specifically, let us consider two collections of paired observations $\psi_{p}={\left\{\left(x_{i}^{p},y_{i}^{p}\right)\right\}}_{i=1}^{M}$ and $\psi_{q}={\left\{\left(x_{j}^{q},y_{j}^{q}\right)\right\}}_{j=1}^{N}$, drawn i.i.d. from joint distributions p(x,y) and q(x,y), respectively. Let $K^{p}$ and $L^{p}$ denote the Gram matrices associated with the variables *x* and *y* under distribution *p*, and similarly let $K^{q}$ and $L^{q}$ denote the corresponding Gram matrices under distribution *q*. Cross-distribution Gram matrices are defined as $K^{pq}\in R^{M\times N}$ and $L^{pq}\in R^{M\times N}$, with entries computed using the same kernel functions.

Under this construction, the conditional CS divergence $D_{\mathrm{CS}}\left(p\left(y|x\right);q\left(y|x\right)\right)$ can be approximated empirically by

(6) $$\begin{array}{cc} {\hat{D}}_{\mathrm{CS}}\left(p\left(y|x\right);q\left(y|x\right)\right) & \approx \log\;\left(\sum_{j=1}^{M}\frac{\sum_{i=1}^{M}K_{ji}^{p}L_{ji}^{p}}{{\left(\sum_{i=1}^{M}K_{ji}^{p}\right)}^{2}}\right)+\log\;\left(\sum_{j=1}^{N}\frac{\sum_{i=1}^{N}K_{ji}^{q}L_{ji}^{q}}{{\left(\sum_{i=1}^{N}K_{ji}^{q}\right)}^{2}}\right)\\ \; & \;-\log\;\left(\sum_{j=1}^{M}\frac{\sum_{i=1}^{N}K_{ji}^{pq}L_{ji}^{qp}}{\left(\sum_{i=1}^{M}K_{ji}^{p}\right)\left(\sum_{i=1}^{N}K_{ji}^{pq}\right)}\right)-\log\;\left(\sum_{j=1}^{N}\frac{\sum_{i=1}^{M}K_{ji}^{qp}L_{ji}^{pq}}{\left(\sum_{i=1}^{M}K_{ji}^{qp}\right)\left(\sum_{i=1}^{N}K_{ji}^{q}\right)}\right). \end{array}$$

This estimator avoids matrix inversion or explicit density ratio estimation. As a result, it scales favorably to high-dimensional settings and serves as a practical building block for conditional distribution-based change detection.

Although CS divergence has been explored in reinforcement learning [13], domain adaptation [26], and shape matching [28], its conditional formulation has not, to our knowledge, been leveraged for interpretable change-point detection.

## 3. Interpretable Change Point Detection via Conditional Cauchy–Schwarz Divergence

### 3.1. Problem Formulation and Framework Overview

We consider an online multivariate time series $X={\left\{x_{t}\right\}}_{t=1}^{\infty }$, where $x_{t}={\left[x_{t}^{1},x_{t}^{2},\ldots ,x_{t}^{m}\right]}^{⊤}\in R^{m}$ denotes observations collected from *m* sensors (or variables) at time *t*. The data stream is generated by an unknown and potentially non-stationary stochastic process, whose underlying distribution may evolve over time.

Given two time instants $t_{1}$ and $t_{2}$ (or two temporal windows centered around them), the goal of change detection is to determine whether the joint data-generating distributions differ, i.e.,

(7) $$P\left(x_{t_{1}}\right)\neq P\left(x_{t_{2}}\right),$$

where $P(x_{t})$ denotes the joint probability distribution of the multivariate observation at time *t*. This formulation does not assume the existence of a fixed steady-state distribution and naturally accommodates both abrupt and gradual distributional shifts in high-dimensional time series. In practice, a change is declared when the estimated divergence exceeds a predefined threshold determined by the desired false alarm level.

Beyond detecting the presence of a change, we are interested in interpreting detected shifts by identifying which variables are most responsible. Let $x^{i}$ denote the *i*-th variable and $x^{-i}=x\setminus \left\{x^{i}\right\}=\left\{x^{1},\ldots ,x^{i-1},x^{i+1},\ldots ,x^{m}\right\}$ denote the collection of all remaining variables. A change is attributed to variable *i* if its conditional distribution with respect to the rest of the system changes across time, namely,

(8) $$P\left(x_{t_{1}}^{i}|x_{t_{1}}^{-i}\right)\neq P\left(x_{t_{2}}^{i}|x_{t_{2}}^{-i}\right).$$

**Figure 1:**
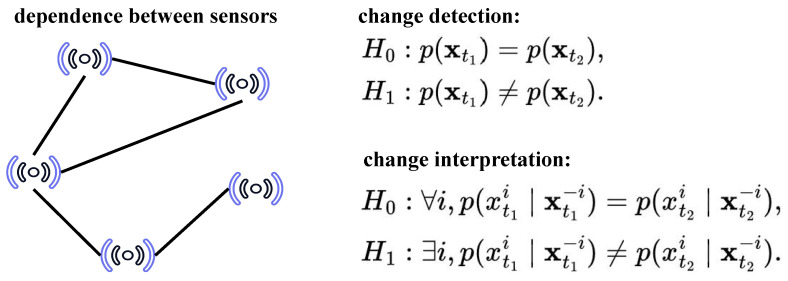
An overview of the proposed change detection and interpretation framework. The key distinction is that detection is performed on joint distributions, while interpretation is achieved via variable-wise conditional analysis. A change alarm is triggered by comparing joint distributions between two time intervals. Conditional distribution shifts are then analyzed at the sensor level to provide post hoc interpretation and localization of change sources.

This conditional perspective captures changes in inter-variable dependency structure and provides a principled notion of interpretability: a variable is deemed responsible for a detected change if its conditional relationship with the rest of the system exhibits a significant distributional shift. Figure 1 illustrates the difference between sensor change detection and change interpretation.

#### Why CS Divergence

As can be seen in Figure 1, our framework, termed Temporal Conditional Divergence (TCD), simultaneously relies on (i) joint distribution comparison for change detection and (ii) conditional distribution comparison for post hoc interpretability. Therefore, the divergence measure must admit both a stable marginal form and a principled conditional extension within a unified functional structure.

While alternative divergences such as KL divergence or MMD could in principle be employed in both stages (e.g., KL with conditional KL, or MMD with conditional MMD), several practical and theoretical limitations arise.

First, commonly used nonparametric estimators of conditional KL divergence, such as *k*-nearest neighbor (k-NN) estimators [29], are not differentiable and may suffer from instability in high-dimensional sliding-window settings. Second, conditional MMD does not admit a universally agreed-upon definition, and its faithfulness depends strongly on the specific formulation of conditional kernel embeddings [30]. Moreover, conditional MMD often requires Gram matrix inversion or regression operators, leading to increased computational complexity [13].

In contrast, the CS divergence admits a unified quadratic formulation for both marginal and conditional distributions [13]. The conditional extension preserves the same Hilbert-space structure as the marginal case, enabling fully nonparametric, differentiable, and inversion-free estimation via kernel Gram matrices. This structural uniformity ensures consistency between detection and interpretation stages and avoids mixing heterogeneous divergence definitions within a single monitoring framework.

Table 1 summarizes key properties of representative conditional divergences. As shown, the conditional CS divergence simultaneously satisfies differentiability, computational feasibility, and faithfulness, making it particularly suitable for interpretable online change detection in multivariate sensor streams.

### 3.2. Window-Based Distribution Comparison

To capture local temporal dynamics and enable online monitoring, we adopt a sliding-window representation of the multivariate time series. Given the data stream $X={\left\{x_{t}\right\}}_{t=1}^{\infty }$ with $x_{t}\in R^{m}$, we define at time index *k* a windowed data matrix

(9) $$X_{k}=\left[x_{k-W+1},\ldots ,x_{k}\right]\in R^{m\times W},$$

where *W* denotes the window length. This construction aggregates consecutive observations and is widely used to approximate local distributions in time series analysis and system monitoring.

At each time step, we compare the distribution induced by observations from a reference window and that from a current window. Let $P(x_{t_{1}})$ and $P(x_{t_{2}})$ denote the empirical distributions estimated from the reference and current windows, respectively. A distributional change is indicated when the discrepancy between these two distributions exceeds a predefined threshold.

This window-based joint distribution comparison is solely used to determine whether a change has occurred. Interpretation and localization of change sources are addressed separately by analyzing conditional distribution shifts at the variable level, as detailed in the next subsection.

### 3.3. Variable-Wise Conditional Change Localization

While joint distribution comparison can indicate whether a distributional change has occurred, it does not reveal which variables are responsible for the detected shift. To enable interpretable change detection, we analyze distributional changes at the level of variable-wise conditional distributions.

At a given time interval, the dependence of $x^{i}$ on the rest of the system is characterized by the conditional distribution $P(x^{i}|x^{-i})$. A change is attributed to variable *i* if this conditional relationship differs across time, i.e., if

(10) $$P\left(x_{t_{1}}^{i}|x_{t_{1}}^{-i}\right)\neq P\left(x_{t_{2}}^{i}|x_{t_{2}}^{-i}\right).$$

To quantify such conditional distribution shifts, we define a variable-wise conditional change score using the conditional CS divergence,

(11) $$S_{i}\left(k\right)=D_{\mathrm{CS}}\;\left(P\left(x_{t_{1}}^{i}|x_{t_{1}}^{-i}\right)\;;\;P\left(x_{t_{2}}^{i}|x_{t_{2}}^{-i}\right)\right).$$

The score $S_{i}\left(k\right)$ measures the extent to which the conditional relationship of variable *i* with respect to the rest of the system has changed. Importantly, the conditional scores ${S_{i}\left(k\right)}_{i=1}^{m}$ are not used to trigger change alarms. They are computed only at detected alarm times and serve as post hoc interpretability measures that decompose a detected distributional shift into variable-wise contributions, enabling fine-grained localization of the sources of change.

### 3.4. Online Change Detection and Post Hoc Interpretation

Combining the joint distribution comparison in Section 3.2 and the conditional change localization in Section 3.3, we obtain a unified framework for online change detection with post hoc interpretability. A generic implementation is summarized in Algorithm 1. The choice of the detection threshold δ is application-dependent. For generic multivariate time series, δ can be set based on empirical statistics or heuristic criteria (e.g., percentile-based thresholds). For industrial process monitoring scenarios, δ can be calibrated from fault-free data to control the false alarm rate, as detailed in Section 4.4.
**Algorithm 1:** Generic Online Change Detection with CS Divergence and Post Hoc Interpretation
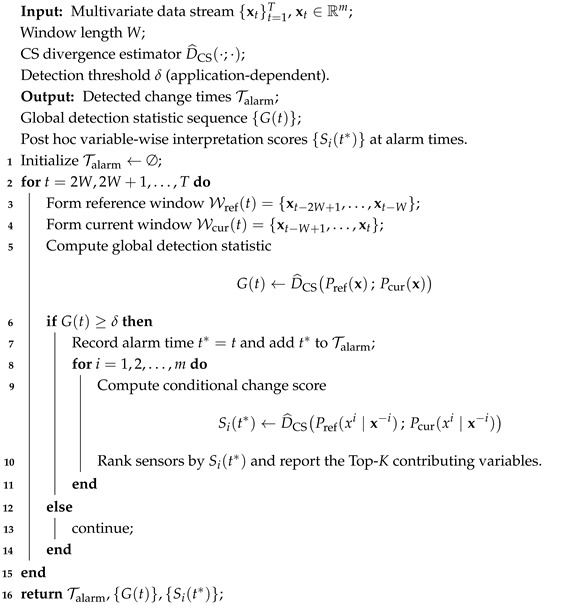


## 4. Experimental Evaluation

This section provides a comprehensive experimental evaluation of the proposed interpretable change detection framework. The experiments are organized progressively from controlled to realistic settings in order to assess detection accuracy, interpretability, and practical applicability. Specifically, we first describe the experimental setup and competing baselines in Section 4.1. We then evaluate the method on synthetic data with known change sources to validate interpretability (Section 4.2), followed by experiments on generic real-world multivariate sensor time series (Section 4.3). Finally, we demonstrate the effectiveness of the proposed framework on a large-scale industrial process benchmark, the Tennessee Eastman Process (TEP) [31,32], to highlight its relevance for industrial sensor monitoring (Section 4.4).

### 4.1. Experimental Setup and Competing Baselines

We compare the proposed method with representative baselines from the literature on multivariate time series change-point detection and distributional change detection. These baselines include kernel-based two-sample tests as well as density-ratio and divergence-based approaches, which are widely adopted in unsupervised detection settings.

All selected baselines produce explicit window-based detection statistics under a unified online evaluation protocol. While several recent works (see Section 2.1) investigate conditional dependence or structural changes, they are not formulated as standalone sliding-window change-point detection algorithms and therefore are not directly comparable within the same experimental framework.

Specifically, we include the following methods:

- MMD [7]: the maximum mean discrepancy-based two-sample test, which detects distributional changes by comparing mean embeddings in a reproducing kernel Hilbert space.
- KDE-based divergence [14]: a KDE-based method that approximates the KL divergence using Rényi’s α-order divergence with α=1.01, following common practice to avoid numerical instability when directly estimating KL divergence [33]. The detailed formulation and KDE-based estimation of Rényi’s α-order divergence are provided in Appendix A.
- RuLSIF [6]: the relative unconstrained least-squares importance fitting method, which detects changes by estimating relative density ratios.
- KL-CPD [9]: a neural network-based change-point detector that maximizes a lower bound of the test power of kernel two-sample tests using an auxiliary generative model.
- NN-CPD [17]: a deep learning–based offline change-point detection framework that trains a neural network classifier to distinguish between sequences with and without change-points.

These baselines are selected to represent different methodological paradigms in distributional change detection, including density-ratio estimation (RuLSIF), kernel-based two-sample testing (MMD), classical divergence estimation (KDE), and neural network–based approaches (KL-CPD, NN-CPD).

For kernel-based methods, including MMD, KDE-based divergence, and the proposed method, we use a Gaussian kernel. The kernel bandwidth σ is selected using the median heuristic, i.e., the median of pairwise distances between samples within each window, which is a widely adopted and parameter-free choice in kernel two-sample testing. This ensures a fair comparison by avoiding dataset-specific manual tuning.

For RuLSIF, the kernel bandwidth is selected via 5-fold cross-validation, and the subspace dimension is set to k=5 with α=0.01, following the recommendations of the original authors. For KL-CPD, the regularization parameter λ is chosen from {0.1,1,10} and the weight β is selected from $\left\{10^{-3},10^{-2},10^{-1},1\right\}$. The best-performing configuration is reported for each dataset.

All methods are evaluated under a unified sliding-window protocol with a stride of 1. The window length *W* is selected from {50,60,…,100} based on validation performance for each dataset. For the generic detection framework (Algorithm 1), the detection threshold δ is determined using a permutation test. Specifically, for each time step, samples from the reference and current windows are randomly permuted to generate surrogate distributions under the null hypothesis of no change. The empirical distribution of the resulting divergence statistics is used to set δ at the 95th percentile, corresponding to a significance level of α=0.05.

All baseline methods are implemented following their original descriptions under the same window configuration and evaluation protocol to ensure fair comparison.

### 4.2. Synthetic Data: Interpretability Study

To validate the interpretability of the proposed method under a controlled setting, we first conduct experiments on synthetic data where the source variables responsible for distributional changes are explicitly known. Unlike real-world datasets, this setting allows us to directly assess whether the detected changes and the associated variable-level attributions are consistent with the underlying data-generating mechanisms.

Following [34], we generate a 5-dimensional multivariate time series consisting of 1900 non-Gaussian samples. Three change points are introduced at time indices [418,980,1411], resulting in four consecutive segments. Within each segment, the data are generated according to a copula Gaussian graphical model [35], denoted by $C_{N}\left(\mu ,K^{-1}\right)$, where the precision matrix *K* encodes the conditional dependency structure among variables. Specifically, an edge between nodes *i* and *j* exists if and only if $K_{ij}\neq 0$.

**Figure 2:**
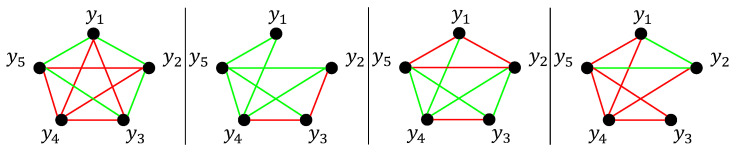
Graph structures in time instants 1–417, 418–979, 980–1410, and 1411–1900 of synthetic time series: red edges indicates positive value in the element of *K*, whereas green edges indicates negative value.

The precision matrix *K* is generated as follows. We first uniformly sample locations ${\left\{y_{i}\right\}}_{i=1}^{m}$ from the unit square and initialize *K* as an identity matrix. For each pair (i,j), the off-diagonal entries ${\left(K\right)}_{ij}={\left(K\right)}_{ji}$ are set to ρ=0.245 with probability ${\left(\sqrt{2\pi }\right)}^{-1}\exp(-4∥y_{i}-y_{j}{∥}^{2})$, and to zero otherwise. The resulting graph structures for each segment are illustrated in Figure 2, where red and green edges indicate positive and negative entries of *K*, respectively.

For each competing method, the detection threshold δ is selected to provide a reasonable trade-off between true positive rate (TPR) and false positive rate (FPR). In addition to detection accuracy, we report the root mean square error (RMSE) between the estimated change-point locations and the ground-truth change points. As summarized in Table 2, the proposed method achieves competitive detection performance compared with existing approaches.

**Figure 3:**
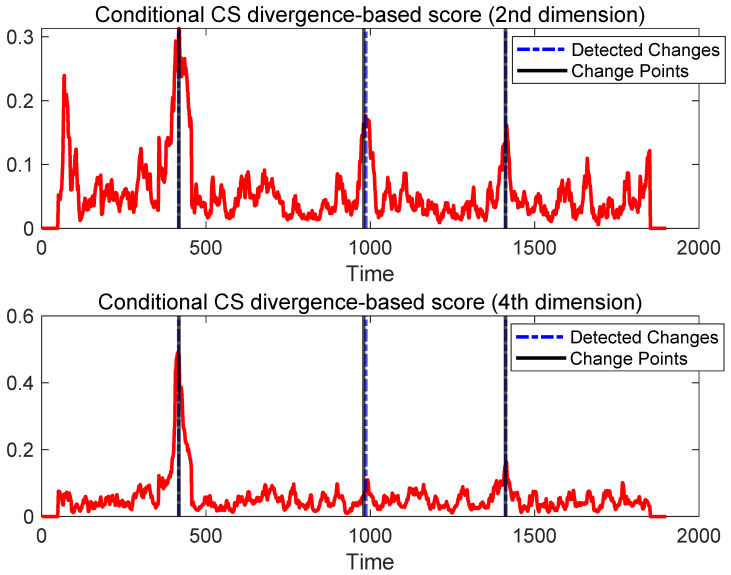
Change point scores on synthetic data obtained using conditional CS divergence for the 2nd dimension (i.e., $p(y_{2}|y_{1},y_{3},y_{4},y_{5})$; top figure) and the 4th dimension (i.e., $p(y_{4}|y_{1},y_{2},y_{3},y_{5})$; bottom figure). Red curve is the obtained score.

More importantly, the synthetic setting enables a fine-grained analysis of interpretability. By examining the variable-wise conditional CS divergence scores $D_{\mathrm{CS}}\;(p_{s}\left(y_{j}|y_{-j}\right)$; $p_{t}\left(y_{j}|y_{-j}\right))$, we observe that different variables contribute unevenly to different change points. For instance, the fourth variable contributes negligibly to the detection of the second change point, which is consistent with the fact that its conditional dependence structure with respect to $\left\{y_{1},y_{2},y_{3},y_{5}\right\}$ remains largely unchanged between segments [418,979] and [980,1410] (see Figure 2). By contrast, the second variable consistently exhibits large conditional divergence scores across multiple change points, reflecting the pronounced alterations in its dependency structure. A visualization of the variable-wise conditional CS divergence is shown in Figure 3.

These results demonstrate that, beyond detecting distributional changes, the proposed method is capable of correctly attributing changes to the underlying variables whose conditional dependencies are truly affected. This controlled experiment provides strong evidence that the conditional CS divergence offers meaningful and reliable interpretability for multivariate change detection.

We further examine the sensitivity of the TCD to the kernel bandwidth parameter. Specifically, we scale the median heuristic by factors of 0.5 and 2.0 to assess robustness under moderate bandwidth variation. As shown in Table 3, the TPR and FPR remain stable across different bandwidth scales. The primary effect is observed in the RMSE of the estimated change-point location, reflecting the expected smoothness–responsiveness trade-off: larger bandwidths produce slightly smoother but delayed detections, whereas smaller bandwidths react faster. Overall, the results indicate that the proposed method is robust to moderate bandwidth perturbations.

### 4.3. Evaluation on Generic Multivariate Sensor Time Series

We further evaluate the proposed method on three widely used real-world benchmarks that represent generic multivariate sensor time series across different modalities, including physiological signals, audio, and video. These datasets have been extensively adopted in the change-point detection literature and allow us to assess the robustness and general applicability of the proposed framework beyond controlled synthetic settings. A summary of the dataset characteristics is provided in Table 4.

The EEG eye state dataset is obtained from the UCI Machine Learning Repository (https://archive.ics.uci.edu/ml/datasets/EEG+Eye+State, accessed on 1 October 2025) and consists of 14,980 time samples recorded from 14 EEG electrodes and one additional reference channel (15 signals in total) over approximately 117 s. The electrodes follow the standard 10–20 EEG placement system as described in the original dataset documentation. No additional preprocessing or dimensionality reduction was applied beyond using the provided normalized signals. Binary eye states (open/closed) were annotated via synchronized video recordings in the original dataset. In our experiments, a change point is defined as a transition between the annotated eye states. Following prior work [36], we evaluate on the first 2000 samples, which contain four abrupt state transitions at time indices 188, 871, 1335, and 1637.

The Great Barbet dataset is constructed following [37] by concatenating birdsong recordings from the same species obtained from the xeno-canto database. Specifically, we download three recordings and concatenate approximately one-minute segments from each song. Each audio frame is represented by 13 mel-frequency cepstral coefficients (MFCCs) along with the log-energy term, resulting in a 14-dimensional feature vector. We use the first 1900, 1800, and 1000 frames from the three recordings, respectively, forming a 14-dimensional time series of length 4700. Change points correspond to transitions between different acoustic regimes.

The UGS dataset [38] is a documentary video from the Open Video Project that contains fast shot transitions and camera motion. The sequence consists of 2143 frames and 11 shots. For each frame, we extract local binary pattern (LBP) features as appearance descriptors, resulting in a 256-dimensional representation. The objective is to detect abrupt shot changes for video temporal segmentation. This dataset has been widely used as a benchmark for change detection in high-dimensional visual time series.

For quantitative comparison on these generic benchmarks, we adopt the receiver operating characteristic (ROC) curve and the area under the ROC curve (AUC), which are commonly used when change-point annotations are available but detection delays may vary across methods. Following prior work [6,7,9], we define the true positive rate (TPR) and false positive rate (FPR) as

(12) $$\left\{\begin{array}{c} \mathrm{TPR}=n_{\mathrm{cr}}/n_{\mathrm{cp}},\\ \mathrm{FPR}=\left(n_{\mathrm{al}}-n_{\mathrm{cr}}\right)/n_{\mathrm{al}}, \end{array}\right.$$

where $n_{\mathrm{cr}}$ denotes the number of correctly detected change points, $n_{\mathrm{cp}}$ is the total number of true change points, and $n_{\mathrm{al}}$ is the total number of detection alarms. Detection alarms are identified as peaks in the change-point score. An alarm at time index *t* is considered correct if there exists a true change point at $t^{*}$ such that $t\in [t^{*}-\tau ,\;t^{*}+\tau ]$, where τ specifies the acceptable detection tolerance.

**Figure 4:**
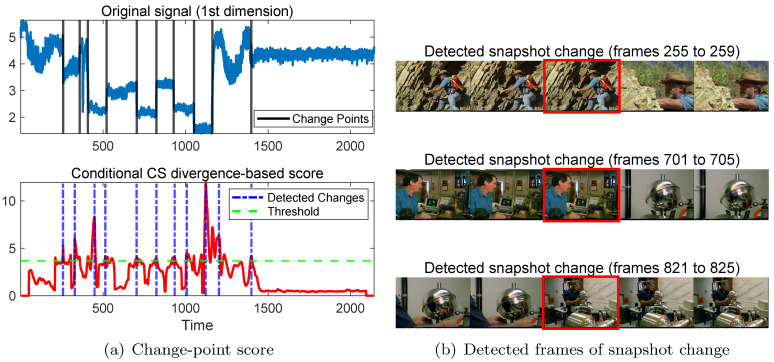
(**a**) Original multivariate UGS signal (first dimension) and corresponding change-point scores obtained by the conditional CS divergence; (**b**) detected frames of snapshot change, marked with red rectangles.

In Table 5, we report the AUC values of all competing methods on the three real-world benchmark datasets. As can be observed, the proposed method consistently achieves the highest AUC across all datasets, indicating superior change detection performance. Figure 4 illustrates the detected change point.

### 4.4. Application to the Tennessee Eastman Process

The experiments in Section 4.3 demonstrate that the proposed method achieves robust change detection performance on a variety of generic multivariate sensor time series across different modalities, including physiological signals, audio, and video. While these benchmarks validate the general applicability of the proposed framework, they do not fully capture the complexity, strong variable coupling, and operational constraints commonly encountered in large-scale industrial process monitoring systems.

To further assess the proposed framework under industrially realistic conditions, we consider the Tennessee Eastman Process (TEP), a widely used benchmark for industrial process monitoring [31,32]. TEP models a large-scale chemical production system with complex nonlinear dynamics, tightly coupled process variables, and diverse fault mechanisms, and has therefore been extensively adopted for evaluating sensor-based fault detection and diagnosis methods.

In this study, we employ the closed-loop TEP simulation model [32,39] to evaluate the proposed change detection and post hoc interpretability framework in an industrial monitoring setting. The process provides 22 continuous process measurements and 11 manipulated variables, resulting in a 33-dimensional multivariate sensor stream. All variables are sampled at a fixed interval of 3 min, following standard TEP configurations commonly used in the process monitoring literature. The detailed monitoring procedure for applying the proposed framework to the Tennessee Eastman Process is summarized in Algorithm 2, which outlines the complete online monitoring pipeline, including threshold calibration from fault-free data, online alarm triggering, and post hoc sensor-level interpretability analysis.

#### 4.4.1. Monitoring Protocol and Threshold Calibration

Rather than adopting a conventional train–test split as in supervised learning, we follow a process monitoring protocol that reflects practical industrial scenarios. Specifically, a continuous segment of fault-free operation is first used as a reference run to characterize normal process behavior. This reference data is used solely for calibrating the detection threshold and does not involve any fault labels.

Let *W* denote the sliding window length. At each time index *t*, two adjacent windows of equal length are constructed from the data stream: a reference window and a current monitoring window. To detect distributional changes in the multivariate sensor stream, we compute a global detection statistic by comparing the joint distributions induced by these two windows using the CS divergence,

(13) $$G\left(t\right)=D_{\mathrm{CS}}\;\left(P_{\mathrm{ref}}\left(x\right);P_{\mathrm{cur}}\left(x\right)\right),$$

where $P_{\mathrm{ref}}\left(x\right)$ and $P_{\mathrm{cur}}\left(x\right)$ denote the empirical joint distributions estimated from the reference and current windows, respectively.
**Algorithm 2:** TEP Monitoring with CS-Divergence Alarming and Conditional CS Interpretability
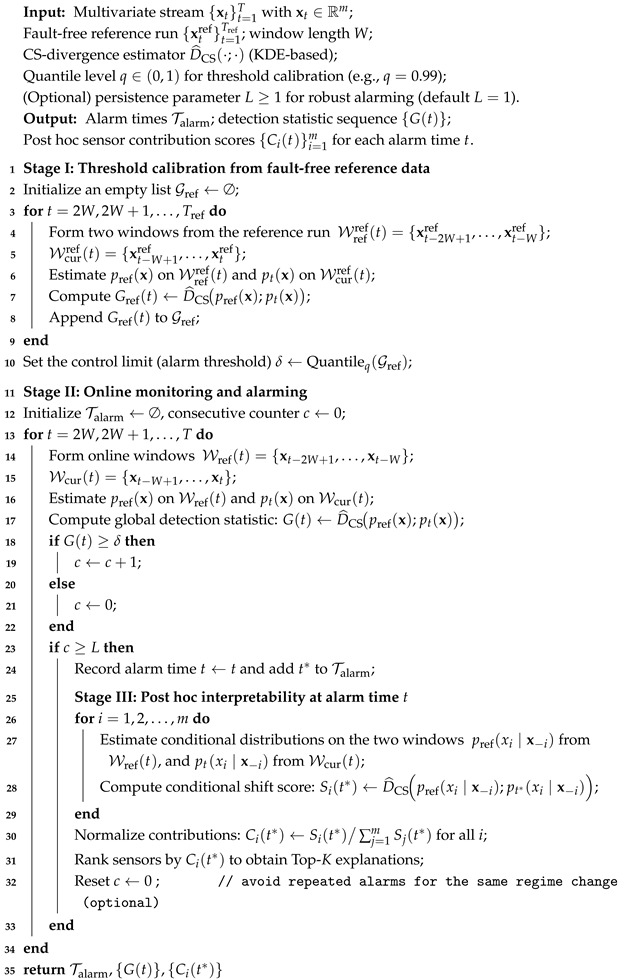


Using the fault-free reference run, we obtain an empirical distribution of G(t) under normal operating conditions and set the alarm threshold δ as a high quantile (e.g., 99%) of this distribution. This threshold serves as a control limit that regulates the false alarm rate and is fixed for all subsequent online monitoring runs.

#### 4.4.2. Online Fault Detection Performance

To evaluate online fault detection performance, we generate independent monitoring runs of 100 h, in which a single fault is injected exactly 20 h after the start of each sequence. This design ensures a sufficiently long fault-free period for assessing false alarms, followed by a clear transition to a faulty operating regime for evaluating detection capability.

Rather than exhaustively evaluating all 21 predefined fault types in the Tennessee Eastman Process (TEP), we focus on a representative subset of commonly studied benchmark faults that span diverse fault mechanisms and detection characteristics. The selected cases include step-type faults (Faults 1–5), a random disturbance (Fault 8), a slow drift fault (Fault 13), and a sticking-related fault (Fault 14). These faults collectively cover abrupt, stochastic, gradual, and nonlinear behaviors, providing broad coverage for assessing both detection accuracy and sensor-level interpretability of the proposed framework.

During online monitoring, an alarm is triggered whenever the detection statistic G(t) exceeds the predefined threshold δ. Detection performance is quantitatively evaluated using three standard metrics widely adopted in industrial process monitoring: the false alarm rate (FAR) and the fault detection rate (FDR). The FAR measures the frequency of spurious alarms under normal operating conditions, the FDR evaluates the proportion of faulty runs in which at least one alarm is raised after fault onset, and Fault Detection Delay (FDD) characterizes the timeliness of detection by measuring the delay between the true fault occurrence time and the first alarm.

Following standard practice in Tennessee Eastman process monitoring, the FAR is computed on fault-free runs, while the FDD is averaged across the selected fault scenarios. The summary results are reported in Table 6. In contrast, due to the heterogeneous detection difficulty of different fault mechanisms, the FDR is reported on a per-fault basis for the selected fault subset, as shown in Table 7. Note that we additionally compare our approach with RTCSA [40], which is widely regarded as a state-of-the-art method for fault change detection.

#### 4.4.3. Post Hoc Sensor-Level Interpretability

Beyond detecting the occurrence of process changes, it is crucial in industrial monitoring applications to understand which sensors and inter-variable dependencies are most responsible for the detected abnormality. Importantly, the proposed framework explicitly decouples change detection from interpretation: sensor-level analysis is performed only after an alarm is triggered and does not affect the alarming mechanism itself. Detailed descriptions of all TEP sensors and manipulated variables are provided in Appendix B.

For interpretability analysis, we focus on Fault 1, which corresponds to a canonical step-type disturbance in feed composition and is among the most well-studied and interpretable fault scenarios in the Tennessee Eastman Process. This fault provides a representative and intuitive test case for examining whether the proposed conditional analysis yields physically meaningful explanations.

Specifically, at the alarm time $t^{*}$, we compute for each sensor *i* the conditional CS divergence

(14) $$S_{i}\left(t^{*}\right)=D_{\mathrm{CS}}\;\left(p_{\mathrm{ref}}\left(x_{i}|x_{-i}\right);p_{t^{*}}\left(x_{i}|x_{-i}\right)\right),$$

which quantifies the change in the conditional dependency between sensor *i* and the remaining sensors. For ease of interpretation, the conditional scores are normalized and used to rank sensors according to their relative contributions to the detected change.

For Fault 1, the top-ranked sensors identified by the proposed method are Sensor 1 (A feed flow), Sensor 2 (D feed flow), and Sensor 8 (reactor level). These sensors are directly associated with upstream feed streams and the resulting reactor operating state, which is consistent with the physical nature of a step-type feed composition disturbance. In particular, changes in the feed flow variables (Sensors 1 and 2) propagate through the process and induce shifts in reactor inventory and dynamics, which are reflected by the reactor level (Sensor 8).

Notably, the prominence of reactor level as a top contributor indicates that the detected change is characterized by altered inter-variable dependency structure rather than isolated marginal deviations in individual sensor readings. This observation highlights the advantage of conditional analysis: while marginal statistics may capture local signal fluctuations, the conditional CS divergence explicitly reveals how relationships between variables evolve under abnormal operating conditions.

Overall, this post hoc analysis demonstrates that the proposed framework not only detects process changes reliably, but also provides physically meaningful and actionable interpretations by localizing the sensors and dependencies most responsible for the detected fault.

## 5. Conclusions and Future Work

In this work, we proposed an interpretable change-point detection framework for multivariate sensor time series based on conditional Cauchy–Schwarz (CS) divergence. By shifting the focus from joint distribution shifts to changes in sensor-wise conditional relationships, the proposed method enables fine-grained attribution of detected changes to specific sensors and their dependencies. The conditional CS divergence can be efficiently estimated in a fully nonparametric manner using kernel density estimation, without density-ratio learning or matrix inversion, making it well suited for online monitoring in high-dimensional settings.

Extensive experiments on synthetic data, generic multivariate benchmarks, and the Tennessee Eastman Process demonstrate that the proposed approach achieves reliable change detection performance while providing transparent, sensor-level interpretations that are not available in conventional CPD methods. In particular, the industrial case study shows that the identified key sensors are consistent with known fault mechanisms, highlighting the practical value of conditional distribution analysis for interpretable monitoring of complex industrial processes.

Beyond the experimental validation presented in this work, the proposed conditional CS divergence-based framework has the potential to support a range of real-world monitoring applications. In large-scale sensor networks and industrial automation systems, it could facilitate early detection of abnormal operating regimes while providing interpretable sensor-level insights into dependency changes. In cybersecurity and network monitoring, conditional distribution analysis may help localize shifts in traffic patterns or feature dependencies, offering improved transparency compared to purely black-box detectors. Similarly, in environmental and structural monitoring contexts, the framework could assist in identifying evolving inter-sensor relationships caused by external disturbances or gradual system degradation.

A natural direction for future work is to extend the current variable-wise attribution framework to explicitly characterize coordinated shifts across subsets of sensors. Although the proposed detection mechanism operates on the full joint distribution and thus captures multivariate changes at the global level, the interpretability module presently evaluates conditional shifts at the level of individual variables. In high-dimensional systems, distributional changes may arise from collective variations among interacting sensors. Extending the conditional CS framework to subset-wise conditioning could enable richer attribution of joint dependency shifts while preserving its distributional, model-agnostic nature.

Moreover, exploring extensions toward directional or causal change analysis, such as integrating transfer entropy measures [41], may further enhance the ability to characterize structural evolution in complex systems.

## Figures and Tables

**Table 1:** Properties of representative conditional divergences. “Diff.” indicates differentiability of the estimator; “Faith.” indicates whether the divergence satisfies the faithfulness property (i.e., D(p(y|x),q(y|x))=0 if and only if p(y|x)=q(y|x)). “✓” indicates that the property is satisfied; “×” indicates that it is not satisfied; and “?” indicates that the property remains unproven.

Method	Hyperparameter	Complexity	Diff.	Faith.
Cond. KL ^1^	*k*	O(kNlogN)	×	✓
Cond. MMD ^2^	kernel size σ, λ	$O(N^{2}d+N^{3})$	✓	? ^3^
Cond. CS (ours)	kernel size σ	$O(N^{2}d)$	✓	✓

^1^ Conditional KL divergence estimated via *k*-nearest neighbor (k-NN) graph. ^2^ *N* denotes the number of samples; *d* denotes the dimensionality of *x* or *y*. ^3^ There is no universally agreed-upon definition of conditional MMD, and its faithfulness depends on the specific formulation.

**Table 2:** Quantitative evaluation in synthetic dataset in terms of true positive rate (TPR), false positive rate (FPR), and the root mean square error (RMSE) between the estimated position of change points and the ground truth. ↑ indicates higher value is better, whereas ↓ indicates smaller value is better.

Method	TPR (↑)	FPR (↓)	RMSE (↓)
RuLSIF	0.33	0.5	2.01
MMD	1	0.5714	7.33
KDE	1	0.7	6.25
KL-CPD	0.66	0.5	3.92
NN-CPD	1	0.5	2.48
TCD (ours)	1	0.4	3.56

**Table 3:** Bandwidth sensitivity analysis for TCD (ours). The bandwidth is scaled relative to the median heuristic. ↑ indicates the higher the better; whereas ↓ indicates the lower the better.

Bandwidth Scale	TPR (↑)	FPR (↓)	RMSE (↓)
0.5×	1.00	0.40	3.38
1.0×	1.00	0.40	3.56
2.0×	1.00	0.40	3.81

**Table 4:** Properties of the datasets (in time series change-point detection).

Datasets	Length	Dimension	Segments
synthetic	1900	5	4
Eye State	2000	15	5
GreatBarbet	4700	14	3
UGS	2143	256	11

**Table 5:** AUC scores on three real-world datasets. The higher value the better performance.

Method	Eye State	Great Barbet	UGS
RuLSIF	0.73	0.67	0.91
MMD	0.79	0.67	0.96
KDE	0.65	0.75	0.95
KL-CPD	0.87	0.88	0.94
NN-CPD	0.85	0.76	0.93
TCD (ours)	0.89	0.92	0.97

**Table 6:** False alarm rates (FARs, %) on fault-free Tennessee Eastman Process data and average fault detection delay (FDD, in samples) on selected TEP faults. The best performance is in bold.

	RuLSIF	MMD	KDE	KL-CPD	NN-CPD	RTCSA	Ours
FAR (%)	2.7	4.4	3.8	3.2	2.9	1.9	**1.2**
FDD (samples)	7.2	11.8	9.6	7.5	8.1	6.4	**5.9**

**Table 7:** Fault Detection Rates (FDRs, %) on selected TEP faults. The best performance is in bold.

Fault ID	RuLSIF	MMD	KDE	KL-CPD	NN-CPD	RTCSA	Ours
Fault 1	97.7	93.3	95.7	97.5	97.5	98.1	**99.0**
Fault 2	95.6	90.3	93.1	95.5	95.3	97.3	**98.5**
Fault 3	92.1	86.3	89.3	92.4	90.9	94.6	**96.8**
Fault 4	90.7	84.4	87.2	90.6	89.7	93.3	**95.2**
Fault 5	92.0	86.5	89.5	92.7	90.1	94.1	**96.4**
Fault 8	86.1	74.2	78.9	87.0	83.8	92.7	**94.0**
Fault 13	80.9	66.7	70.0	82.2	77.5	88.0	**91.0**
Fault 14	98.0	94.8	96.9	98.5	98.0	99.4	**99.5**

## Data Availability

The data used in this study are publicly available. The EEG Eye State dataset is available from the UCI Machine Learning Repository. The Great Barbet audio recordings were obtained from the xeno-canto database. The UGS video dataset is available from the Open Video Project. The Tennessee Eastman Process data were generated using the standard simulation model under commonly adopted configurations. All data sources are cited in the manuscript, and no new data were created.

## Abbreviations

The following abbreviations are used in this manuscript:

CS	Cauchy–Schwarz
CPD	Change point detection
FAR	False Alarm Rate
FDR	Fault Detection Rate

## Appendix A. KDE-Based Rényi Divergence Estimation

In this appendix, we briefly review the kernel density estimation (KDE)-based approximation of Rényi’s α-order divergence, which is used as a baseline method in the experimental evaluation.

Rényi’s α-order divergence of g(x) from f(x) is defined as

(A1) $$D_{\alpha }\left(f;g\right)≜\frac{1}{\alpha -1}\log\int f\left(x\right){\left(\frac{f(x)}{g(x)}\right)}^{\alpha -1}dx.$$

### Appendix A.1. Limiting Case α→1: Connection to KL Divergence

Applying L’Hôpital’s rule to Equation (A1) yields

(A2) $$\begin{array}{cc} \lim_{\alpha \to 1}D_{\alpha }\left(f;g\right) & =\lim_{\alpha \to 1}\frac{1}{\alpha -1}\log\int f\left(x\right){\left(\frac{f(x)}{g(x)}\right)}^{\alpha -1}dx\\ \; & =\frac{\lim_{\alpha \to 1}\int -f\left(x\right){\left(\frac{f(x)}{g(x)}\right)}^{\alpha -1}\log\;\left(\frac{g(x)}{f(x)}\right)dx}{\lim_{\alpha \to 1}\int {\left(\frac{f(x)}{g(x)}\right)}^{\alpha -1}dx}\\ \; & =\int f\left(x\right)\log\;\left(\frac{f(x)}{g(x)}\right)dx\\ \; & =D_{\mathrm{KL}}\left(f;g\right), \end{array}$$

which shows that Rényi’s α-order divergence converges to the Kullback–Leibler (KL) divergence as α→1.

### Appendix A.2. KDE-Based Nonparametric Estimation of Rényi Divergence

Direct estimation of the Kullback–Leibler divergence using kernel density estimation (KDE) is known to be numerically unstable in high-dimensional settings. To address this issue, a common practice is to approximate the KL divergence using Rényi’s α-order divergence with α chosen close to 1.

Let ${\left\{x_{i}^{f}\right\}}_{i=1}^{N}$ and ${\left\{x_{i}^{g}\right\}}_{i=1}^{M}$ be *i.i.d.* samples drawn from densities f(x) and g(x), respectively. Using KDE, the densities are estimated as

(A3) $$\hat{f}\left(x\right)=\frac{1}{N}\sum_{i=1}^{N}G_{\sigma }\left(x-x_{i}^{f}\right),\;\hat{g}\left(x\right)=\frac{1}{M}\sum_{i=1}^{M}G_{\sigma }\left(x-x_{i}^{g}\right),$$

where $G_{\sigma }\left(\cdot \right)$ denotes a Gaussian kernel with bandwidth σ.

The Rényi divergence can be expressed as

(A4) $$D_{\alpha }\left(f;g\right)=\frac{1}{\alpha -1}\log E_{f}\left[{\left(\frac{f(x)}{g(x)}\right)}^{\alpha -1}\right],$$

which can be approximated empirically by

(A5) $$\begin{array}{cc} D_{\alpha }\left(f;g\right) & \approx \frac{1}{\alpha -1}\log\frac{1}{N}\sum_{j=1}^{N}{\left(\frac{\hat{f}\left(x_{j}^{f}\right)}{\hat{g}\left(x_{j}^{f}\right)}\right)}^{\alpha -1}\\ \; & =\frac{1}{\alpha -1}\log\frac{1}{N}\sum_{j=1}^{N}{\left(\frac{\sum_{i=1}^{N}G_{\sigma }\left(x_{i}^{f}-x_{j}^{f}\right)}{\sum_{i=1}^{M}G_{\sigma }\left(x_{i}^{g}-x_{j}^{f}\right)}\right)}^{\alpha -1}. \end{array}$$

This estimator follows the standard KDE-based approximation of Rényi’s divergence and is adopted as a baseline method in the experimental evaluation.

## Appendix B. Monitoring Variables in the Tennessee Eastman Process

The TEP benchmark provides a set of continuous process measurements and manipulated variables that characterize the operating conditions of a large-scale chemical production system. In this work, we adopt the standard TEP variable notation commonly used in the process monitoring literature, where variables are indexed by prefixes indicating their physical types, including flow-related variables (F), temperatures (T), pressures (P), levels (L), and manipulated variables (V).

Table A1 summarizes the definitions of all monitoring variables used in our experiments, together with their corresponding physical meanings. This table is provided to facilitate the interpretation of the sensor-level results reported in Section 4.4, particularly for the post hoc analysis of conditional dependency changes under different fault scenarios.

**Table A1:** Monitoring variables in the Tennessee Eastman Process (TEP).

Variable	Description	Variable	Description
F1	A feed (stream 1)	T18	Stripper temperature
F2	D feed (stream 2)	T19	Stripper steam flow
F3	E feed (stream 3)	C20	Compressor work
F4	A and C feed (stream 4)	T21	Reactor cooling water outlet temperature
F5	Recycle flow (stream 8)	T22	Separator cooling water outlet temperature
F6	Reactor feed rate (stream 6)	V23	D feed flow (stream 2)
P7	Reactor pressure	V24	E feed flow (stream 3)
L8	Reactor level	V25	A feed flow (stream 1)
T9	Reactor temperature	V26	A and C feed flow (stream 4)
F10	Purge rate (stream 9)	V27	Compressor recycle valve
T11	Product separator temperature	V28	Purge valve (stream 9)
L12	Product separator level	V29	Separator pot liquid flow (stream 10)
P13	Product separator pressure	V30	Stripper liquid product flow (stream 11)
F14	Product separator underflow (stream 10)	V31	Stripper steam valve
L15	Stripper level	V32	Reactor cooling water flow
P16	Stripper pressure	V33	Condenser cooling water flow
F17	Stripper underflow (stream 11)		
